# Habitat suitability maps for juvenile tri-spine horseshoe crabs in Japanese intertidal zones: A model approach using unmanned aerial vehicles and the Structure from Motion technique

**DOI:** 10.1371/journal.pone.0244494

**Published:** 2020-12-23

**Authors:** Akihiko Koyama, Taiga Hirata, Yuki Kawahara, Hiroki Iyooka, Haruka Kubozono, Norio Onikura, Shinji Itaya, Tomoko Minagawa

**Affiliations:** 1 Faculty of Advanced Science and Technology, Kumamoto University, Kurokami, Chuo-ku, Kumamoto, Japan; 2 Department of Civil and Environmental Engineering, Kumamoto University, Kurokami, Chuo-ku, Kumamoto, Japan; 3 Department of Civil Engineering, Fukuoka University, Nanakuma, Jonan-ku, Fukuoka, Japan; 4 The 21st Century Program, Kyushu University, Motooka, Nishi-ku, Fukuoka, Japan; 5 Fishery Research Laboratory, Kyushu University, Tsuyazaki, Fukutsu, Japan; 6 Tsuyazaki Seaside Nature School, Tsuyazaki, Fukutsu, Japan; Tanzania Fisheries Research Institute, UNITED REPUBLIC OF TANZANIA

## Abstract

The tri-spine horseshoe crab, *Tachypleus tridentatus*, is a threatened species that inhabits coastal areas from South to East Asia. A Conservation management system is urgently required for managing its nursery habitats, i.e., intertidal flats, especially in Japan. Habitat suitability maps are useful in drafting conservation plans; however, they have rarely been prepared for juvenile *T*. *tridentatus*. In this study, we examined the possibility of constructing robust habitat suitability models (HSMs) for juveniles based on topographical data acquired using unmanned aerial vehicles and the Structure from Motion (UAV-SfM) technique. The distribution data of the juveniles in the Tsuyazaki and Imazu intertidal flats from 2017 to 2019 were determined. The data were divided into a training dataset for HSM construction and three test datasets for model evaluation. High accuracy digital surface models were built for each region using the UAV-SfM technique. Normalized elevation was assessed by converting the topographical models that consider the tidal range in each region, and the slope was calculated based on these models. Using the training data, HSMs of the juveniles were constructed with normalized elevation and slope as the predictor variables. The HSMs were evaluated using the test data. The results showed that HSMs exhibited acceptable discrimination performance for each region. Habitat suitability maps were built for the juveniles in each region, and the suitable areas were estimated to be approximately 6.1 ha of the total 19.5 ha in Tuyazaki, and 3.7 ha of the total 7.9 ha area in Imazu. In conclusion, our findings support the usefulness of the UAV-SfM technique in constructing HSMs for juvenile *T*. *tridentatus*. The monitoring of suitable habitat areas for the juveniles using the UAV-SfM technique is expected to reduce survey costs, as it can be conducted with fewer investigators over vast intertidal zones within a short period of time.

## Introduction

Horseshoe crabs are a type of estuarine chelicerate arthropods (Chelicerata) that are known as “living fossils” because they have existed for over 200 million years [1]. There are four extant species of horseshoe crabs: *Limulus polyphemus* (Linnaeus, 1758), *Tachypleus tridentatus* (Leach, 1819), *Tachypleus gigas* (OF Müller, 1785), and *Carcinoscorpius rotundicauda* (Latreille, 1802). Horseshoe crabs are an important topic of discussion in the field of evolutionary ecology [2,3]. Moreover, the blood of horseshoe crabs is commercially used in medicine, and their eggs are an important food source for migratory shore birds and eels [4,5]. They are also useful as a flagship species to spread public awareness regarding biodiversity conservation [6]. However, their habitats are continually diminishing worldwide due to anthropogenic activities, and potentially due to the sea level rise induced by climate change [7]. Thus, the conservation of horseshoe crabs is ecologically and commercially important, and it is now receiving increased global attention [6,8–10]. Progress in this field of study may contribute to the sustainable management of estuarine ecosystems.

The tri-spine horseshoe crab, *Tachypleus tridentatus*, is distributed across the countries in South and East Asia, such as Indonesia, Vietnam, the Philippines, Malaysia, Hong Kong, China, Taiwan, and Japan [11,12]. This species spawns in the upper intertidal zones of sandy beaches, and intertidal flats covered with fine sediment serve as nurseries for the juveniles. In Japan, juveniles with a prosomal width of up to 7.0 cm are found in intertidal flats, while the larger individuals inhabit the subtidal zones; i.e., sub-adults and adults inhabit the offshore regions [13–15]. Their nursery and spawning habitats, i.e., intertidal flats and sandy beaches, are vulnerable to anthropogenic disturbances [16,17], which may cause habitat loss and further reduce the population of this species. In fact, the conservation status of this species was changed from “Data Deficient (DD)” to “Endangered (EN)” in the IUCN Red List in 2018 [12]. In Japan, which is the northernmost recorded habitat of this species, there were hundreds of thousands of individuals of this species until the early 20th century; however, the total population is estimated to have declined to less than 10,000 in recent years [15]. More than 40% of the estuarine environments in Japan disappeared between 1945 and 2005 due to anthropogenic impacts [18,19], and this is one of the reasons for the decrease in the *T*. *tridentatus* population [15]. As a result, this species has been regarded as a threatened species by the Ministry of the Environment since 2006 [12]. Therefore, to address the declining population of juvenile *T*. *tridentatus*, conservation and restoration of intertidal flats are urgently required in Japan.

The habitat characteristics of juvenile *T*. *tridentatus* have been examined in several studies. For example, several environmental factors, such as elevation, mud, water, and organic matter content in sediment, and the density of prey have the potential to affect the distribution of juveniles [6,20–22]. Conversely, salinity, dissolved oxygen, and water temperature are considered to be non-critical factors [23]. The construction of habitat suitability models (HSMs) using these environmental factors can support conservation planning and enable the selection of effective conservation and restoration areas through mapping [24–26]. However, the habitat suitability of this species has only been estimated at a global scale [11]. At the local scale, the population density of juveniles has been estimated based on the sampling areas of each study [20,22,27–29]. One reason for the lack of surveys covering wide areas is that the duration of low tide, during which surveying is possible, is very limited. Further, muddy sediment, which is the main habitat of juveniles, is difficult to traverse.

In recent years, the integration of small unmanned aerial vehicles and the Structure from Motion (UAV-SfM) technique have facilitated the construction of topographical models at high spatial resolutions [30]. Several studies have reported that the microtopography of intertidal zones, including tidal flats, can be recreated with an error of less than 10 cm [31–33]. The UAV-SfM technique is expected to be effective for habitat suitability mapping in intertidal zones, as it can acquire images of vast intertidal zones within a short time frame. For example, this technique has been shown to simplify the mapping of salt marsh plants [33], but it has rarely been applied to macrobenthos.

Iyooka [34] constructed an HSM for juvenile *T*. *tridentatus* in the Tsuyazaki intertidal flats, one of the habitats of this species in Japan. They used the topographical data acquired using the UAV-SfM technique. Although an HSM should be robust in predictive accuracy [35,36], Iyooka [34] did not verify their HSM’s robustness. In the present study, we constructed HSMs using the data obtained through a survey along with the data used by Iyooka [34]. To test the robustness of the HSM, an evaluation was performed using external data collected by several scientists and citizen scientists in Tsuyazaki. Furthermore, we collected the distribution data of the juveniles and built a topographic model of an intertidal zone using the UAV-SfM technique in another survey area, the Imazu tidal flats, in Japan. Our study aimed to examine the possibility of constructing a robust HSM for juvenile *T*. *tridentatus* with topographical data acquired using the UAV-SfM technique, and to create habitat suitability maps of the Tsuyazaki and Imazu intertidal flats.

## Material & methods

### Survey area

**Fig 1:**
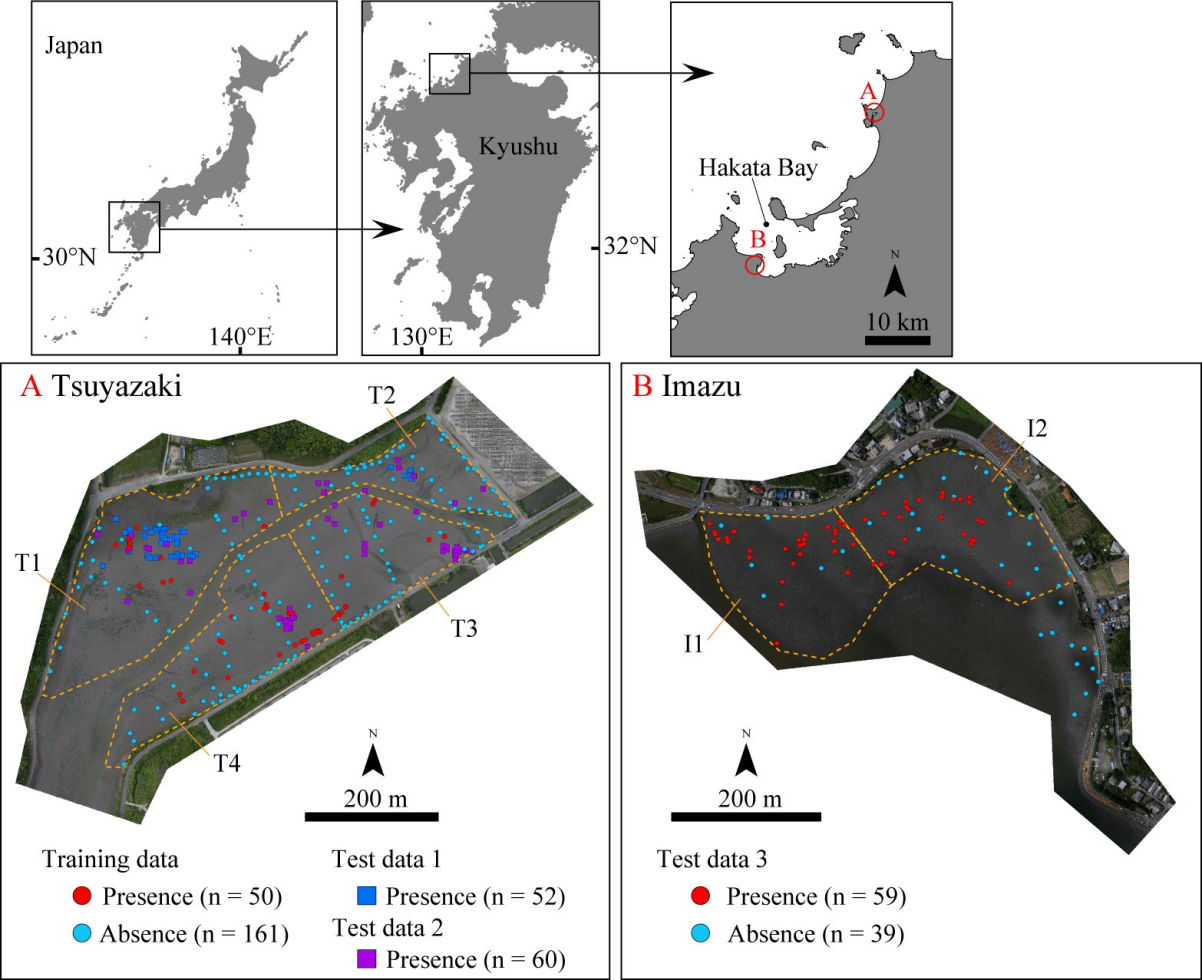
Maps of survey areas in Tsuyazaki and Imazu intertidal flats. Coastline data were downloaded from the National Land Numerical Information download service (https://nlftp.mlit.go.jp/ksj/index.html). Republished from the coastline data under a CC BY license, with permission from National Land Information Division, National Spatial Planning and Regional Policy Bureau, MLIT of Japan, original copyright 2020. Orthophoto of Tsuyazaki and Imazu were constructed using the SfM based on photographs taken by us.

In the present study, we targeted the Tsuyazaki tidal flats as a survey area to construct an HSM (Fig 1). These intertidal flats have a circumference of approximately 4 km and are located in an inlet in Fukutsu City, Japan. The tidal range near Tsuyazaki is approximately 1.7 m during the spring tide (Japan Coast Guard: https://www1.kaiho.mlit.go.jp/KANKYO/TIDE/datum/). Sandy and/or muddy intertidal zones extend inside the inlet [37,38], and a cumulative total of more than 1500 juvenile *T*. *tridentatus* individuals have been found there [39]. Several studies have been conducted in Tsuyazaki on this species, including studies for migration patterns [38] and monitoring for spawning [40,41], suggesting that this intertidal area is reasonable for our work.

To test the robustness of the HSM, we also studied the Imazu tidal flats located in Hakata Bay, Fukuoka City (Fig 1). The tidal range in Hakata Bay is approximately 2.2 m (Japan Coast Guard: https://www1.kaiho.mlit.go.jp/KANKYO/TIDE/datum/). The highest spawning rate of *T*. *tridentatus* in Hakata Bay has been observed in the Imazu intertidal zones [14,42]. This is also the main habitat for the juveniles. Several local governments in Japan have ordinances for protection of this species or its habitats. However, due to an absence of ordinance regulation for this species in the Fukuoka Prefecture, no permits were required for our study. In our surveys, all the juveniles were treated gently and were immediately released in the locations at which they were found.

### Distribution data sampling for HSM construction

The winter months were excluded from the surveys for HSM construction and evaluation, considering the hibernation period of this species [14,43]. For HSM construction, the presence/absence data for the juveniles were obtained from the survey areas in Tsuyazaki in May–August 2018 and July 2019 for a total of eight days. These months were selected because it is during this period that the juveniles are most active. The surveys were conducted in the day time during low tide.

First, the absence data were obtained in May within one day. In the survey area, 110 points were set at intervals of approximately 15–30 m, and their latitudes, longitudes, and elevations were measured using a Real Time Kinematic-Global Navigation Satellite System (RTK-GNSS; Trimble R4 GNSS, Nikon-Trimble Co., Ltd.). Elevation was assessed relative to Tokyo Peil (T.P.). The horizontal and vertical position errors of this measurement system are approximately a few centimeters. The main purpose of this measurement survey was to grasp the intertidal topography of the area; however, no juveniles were visually observed within 1 m of each point. This survey was conducted 2 h before and after the low tide by three investigators, including the first and second authors.

Next, the presence data for the juvenile *T*. *tridentatus* were collected over six days between July and August 2018, and on one day in July 2019. Each survey was conducted 1 h before and after the low tide during spring tide, totaling a survey time of 14 h. The survey area was divided into four sections, T1, T2, T3, and T4, and the survey time allocated for T1 and T2 was 3 h each, while that for T3 and T4 was 4 h each (Fig 1 and S1 Fig). The walk-through survey strategy was adopted due to its effectiveness in collecting juvenile data [27]. With this strategy, two investigators (first and second/third authors) lined up side-by-side at intervals of 5 to 10 m and walked from high to low tide level within each survey area. Subtidal zones were not surveyed. The latitude, longitude, and elevation of each point where the juveniles were present were measured using RTK-GNSS. The collected juveniles were photographed along with a scale bar, and their prosomal widths were measured to the nearest 0.1 cm using the photographs and ImageJ software (ver. 1.52t) [44]. The prosomal widths were in the range of 1.1–6.5 cm, with an average of 3.8 ± 1.6 cm (n = 50). In this manner, the presence data were acquired from 50 points.

Lee and Morton [45] reported that juvenile *T*. *tridentatus* bury and hide at a depth of approximately 3 cm from the sediment surface. Thus, for all the points where juveniles appeared, surface sediments up to a depth of 3 cm were collected using 45 mL plastic tubes with a diameter of 3 cm. The sediment samples were weighed after collection (wet weight), after drying, and after sieving with a 0.063 mm sieve. The water weight of each sediment sample was calculated by the difference between the wet weight and the dry weight, and the percentage of the water weight to the wet weight was assessed as water content. The weight of silt & clay (grain size < 0.063 mm) was calculated by the difference between the dry weight and the weight after sieving, and the percentage of silt & clay to the dry weight was assessed as mud content.

### Test data for HSM evaluation

Three external datasets were used for the HSM evaluation. Two of these were based on surveys in Tsuyazaki conducted from 2017 to 2019, while the third dataset was based on surveys in the Imazu tidal flat in 2019. These surveys were conducted in daytime during the low tide.

The distribution data of juvenile *T*. *tridentatus* were mainly collected by the fifth and sixth authors in Tsuyazaki over four days between July and August 2017. The surveys were conducted 2 h before and after the low tide during spring tide, totaling a survey time of 16 h. Surveys were conducted by six investigators, who mainly covered two survey areas, T1 and T2. The latitude and longitude for the collection points of the juveniles were recorded using handy GPS devices (eTrex® 20J and eTrex® 30J, Garmin Ltd.). The collected juveniles were released at each collection point after marking their dorsal side. Through these surveys, presence data were obtained from 55 points. The marked juveniles were found at three of these presence points. Prosomal width was measured for some of the collected juveniles, and it was found to range from 2.2 to 6.3 cm, with an average of 3.7 ± 1.9 cm (n = 35).

Furthermore, in Tsuyazaki, the distribution data of the juveniles were mainly obtained by the seventh author over three days between May and August 2018, and two days between July and September 2019. The surveys were conducted 2 h before and after the low tide during spring tide, totaling a survey time of 20 h. These surveys were conducted in the survey areas by a team of 11–16 investigators (13.8 ± 2.2, n = 5). Each investigator walked freely on the survey areas to observe the juveniles based on the previously described survey method [39]. The latitude and longitude for the collection points of the juveniles were recorded using a handy GPS (eTrex® 20J, Garmin Ltd.). Presence data were obtained from 60 points. The author prepared the survey sheets diagrammatically, showing the range of the prosomal width from the third to the eighth instar stages based on the studies by Sekiguchi [14] and Wada et al. [39]. In their surveys, the instar stage of each collected juvenile was estimated using survey sheets, covering a range from the third to the eighth instar stages.

Finally, the distribution data of the juveniles in the Imazu tidal flats were obtained over two days in September 2019. The surveys were conducted 1 h before and after the low tide during spring tide, totaling a survey time of 4 h. The survey area was divided into two sections, I1 and I2, each of which was surveyed by the first and third authors for 2 h (Fig 1). The latitude, longitude, and elevation for the collection points of the juveniles were measured using RTK-GNSS. The collected juveniles were photographed along with a scale bar, and their prosomal widths were measured to the nearest 0.1 cm from the photo. Through the surveys, presence data were obtained from 59 points. The prosomal width ranged between 2.3–7.2 cm, with an average of 4.3 ± 1.2 cm (n = 59). The surface sediments up to a depth of 3 cm were collected at points where the juveniles appeared using 45 mL plastic tubes with a diameter of 3 cm. However, the sediments could not be collected at 12 of the 59 points due to an insufficient number of plastic tubes. The collected sediments were then measured for determination of water and mud content.

### UAV-SfM

Aerial photography was performed using a small UAV (DJI F550-N3, Da-Jiang Innovations Science and Technology Co., Ltd.) in the Tsuyazaki intertidal flats approximately 30 min before and after the low tide during spring tide in June 2019. The camera and lens used were EOS-M and EF-M22mm F2 STM (Canon Inc.), respectively, and they were attached under the UAV using a Picavet according to the method described in Inoue et al. [46]. The flight speed and elevation were set to 6 m/s and 100 m above mean sea level, respectively. The camera images were captured every 2 s at 2 cm ground resolution, and the overlapping of successive aerial photographs was more than 80%. In the Imazu intertidal flats, aerial photography was performed approximately 30 min before and after the low tide during spring tide in July 2019. The flight and photographical conditions were the same as those used in Tsuyazaki. The flight control of the UAV was performed using the application DJI GS PRO (Da-Jiang Innovations Science and Technology Co., Ltd.), and the UAV was operated by the fourth author.

Ground control points (GCPs) and ground references (GRs) are required to recreate the topographical model with a high accuracy using the UAV-SfM technique. The GCPs are used to define the coordinate reference system and to create a high quality digital surface model (DSM), while the GRs are used to assess the quality of the DSM [32]. Before conducting aerial photography at Tsuyazaki and Imazu, the latitude, longitude, and elevation at 64 points for GCPs and GRs were measured using RTK-GNSS over a wide area in each region by the first and third authors (S2 Fig). At the same time, the presence/absence of juvenile *T*. *tridentatus* was checked within a radius of 1 m from the points set on the intertidal flats. The GCPs and GRs without juveniles were used as the absence data to construct the HSM and test model robustness.

DSMs were created using 275 and 350 aerial photographs for Tsuyazaki and Imazu, respectively. The cell size was set to 0.2 m. For the DSM creation, 31 and 32 GCPs were set at Tsuyazaki and Imazu, respectively (S2 Fig). The DSMs were created using SfM software (Agisoft Metashape Professional Edition, Agisoft LLC).

It has been reported that the effects of sun glint on water surfaces degrades the accuracy of DSM generation [32]. In fact, several raster cells of the subtidal zone of Tsuyazaki, covering the rill part, showed unrealistic elevation (S3 Fig). This error might affect the construction of the HSM. Therefore, 264 points of the rill were measured using RTK-GNSS in September 2019, and the rill bottom elevation of DSM was rectified according to the interpolated data using the Natural Neighbor tool of Spatial Analyst in ArcGIS 10.6 (S3 Fig). The DSM of Imazu also showed unrealistic values of the subtidal zones. However, these zones were excluded from the analysis area. Consequently, the analyzed areas of Tsuyazaki and Imazu were approximately 19.5 and 7.9 ha, respectively.

**Fig 2:**
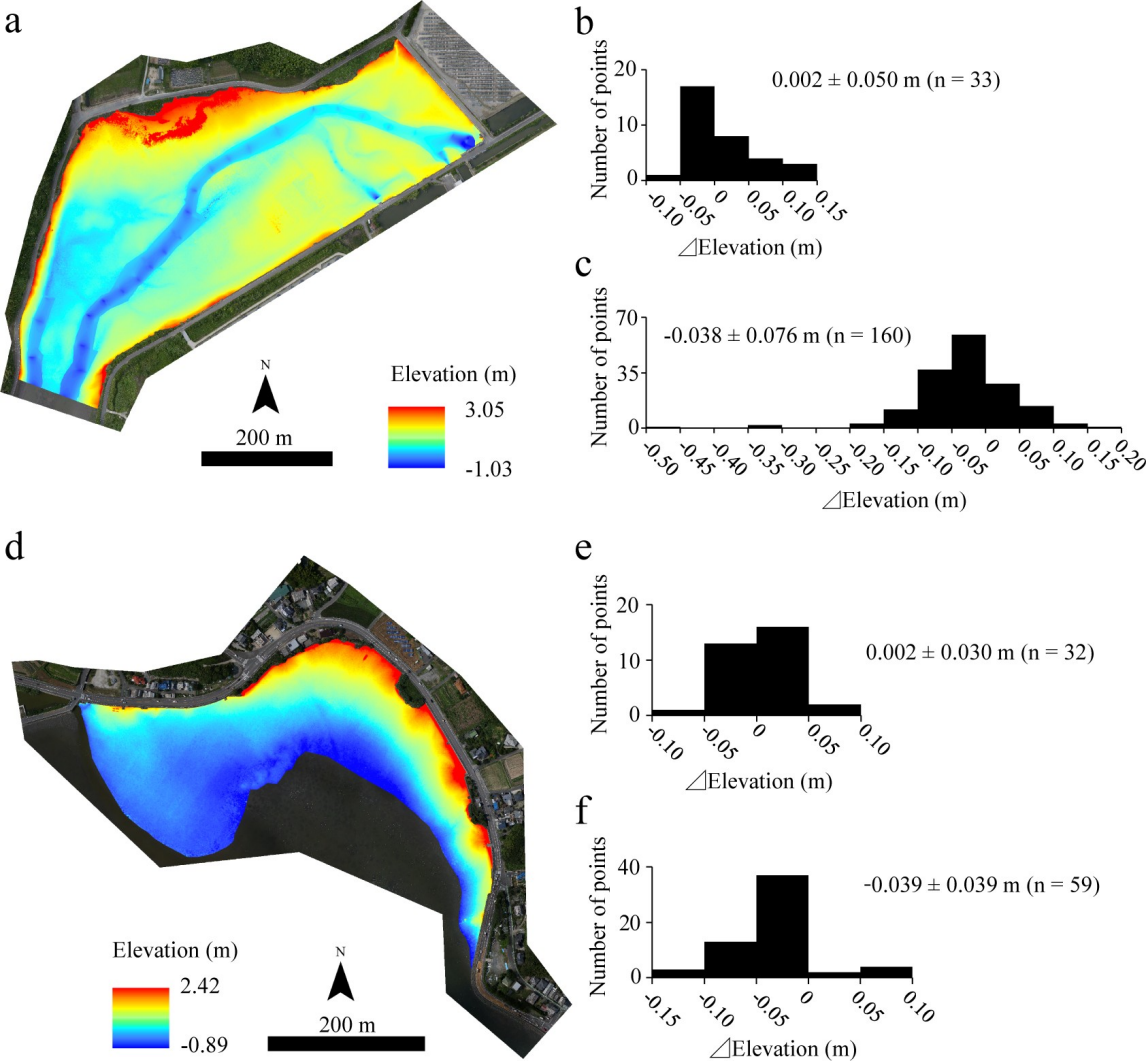
Elevation estimated using digital surfaces models (DSMs) for Tsuyazaki (a) and Imazu (d) tidal flats. Performances of the models were evaluated according to ground references (b and e), and presence/absence points of juvenile horseshoe crabs (c and f). The orthophotos (a and d) were constructed using the SfM based on aerial photographs taken by us.

The elevations estimated by the DSMs for the analyzed areas in the Tsuyazaki and Imazu intertidal flats are shown in Fig 2 and S2 File. The vertical errors between DSM and GR (⊿Elevation) were 0.002 ± 0.050 m (mean ± standard deviation, n = 33) in Tsuyazaki, and 0.002 ± 0.030 m (n = 32) in Imazu. This implies that each DSM showed a high accuracy. The vertical errors were also assessed between DSM and the presence/absence points (n = 50 and 110, respectively) because there was a gap of approximately one year between the juvenile survey and aerial photography at Tsuyazaki. The error was found to be −0.038 ± 0.076 m (n = 160), which is not sufficiently high, although it lies in the range −0.48–0.15 m. Conversely, the error between the DSM and presence points in Imazu was −0.039 ± 0.039 m (n = 59), which is very small. Therefore, the DSMs were judged to be usable for the HSM construction.

### Environmental factors

**Fig 3:**
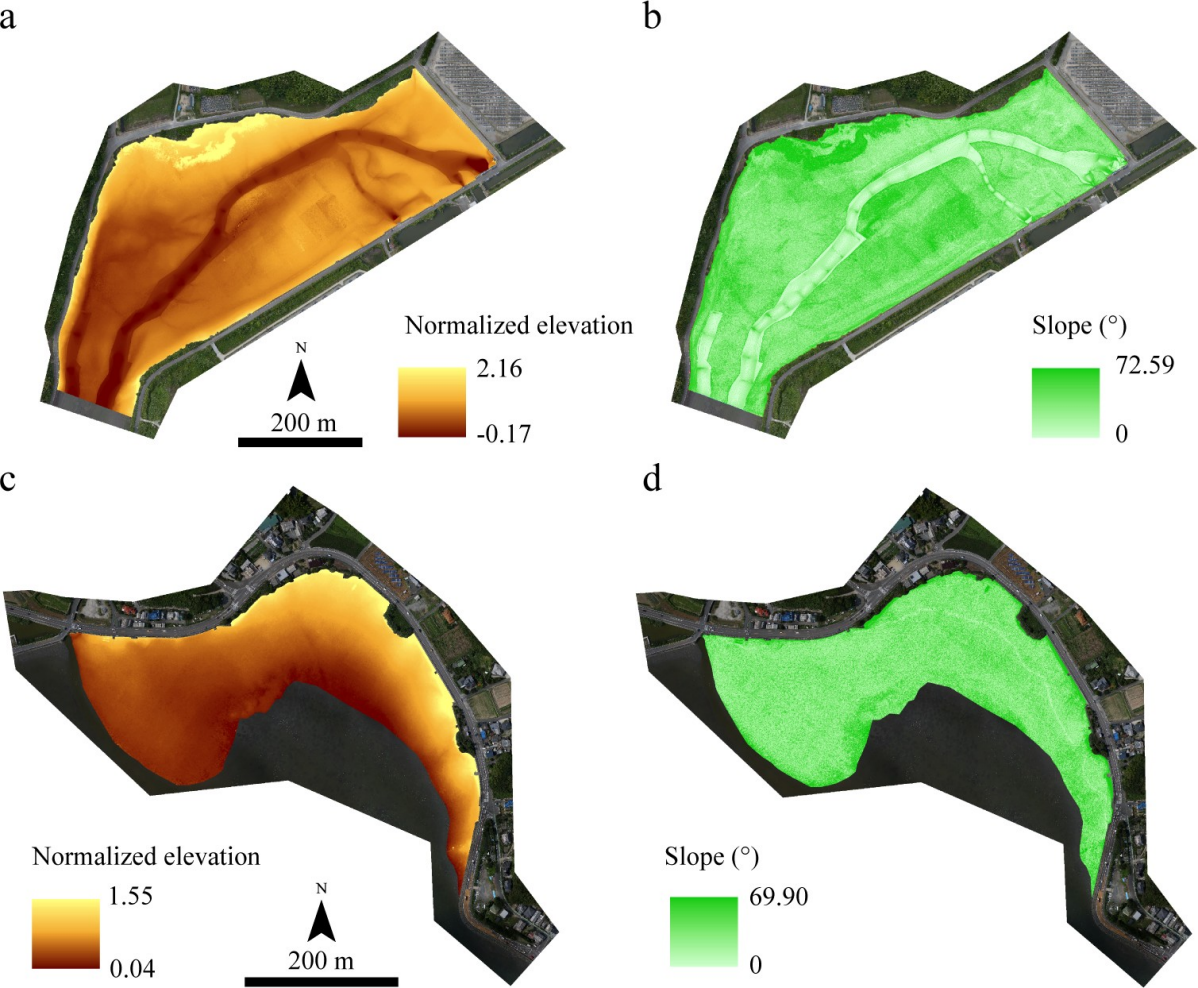
Normalized elevation and slope in the survey area of Tsuyazaki (a and b) and Imazu (c and d) tidal flats. The orthophotos were constructed using the SfM based on aerial photographs taken by us.

The elevation was converted based on each tidal range because the tidal ranges of the Tsuyazaki and Imazu tidal flats were different. In the present study, the DSM was converted to normalized values by setting the elevation of the low and high tide lines to 0 and 1, respectively. Thus, this variable was regarded as the normalized elevation (Fig 3). The nearest tidal observation points in Tsuyazaki and Imazu are Ashiya (33°54′ N, 130°40′ E) and Hakata-Higashihama (33°78′06″ N, 130°24′31″ E), respectively (Japan Coast Guard: https://www1.kaiho.mlit.go.jp/KANKYO/TIDE/datum/). The elevations of low and high tide lines in Ashiya are T.P. −0.72 m and T.P. +1.02 m, respectively, while those in Hakata-Higashihama are T.P. −0.99 m and T.P. +1.21 m, respectively. For example, when the elevation is T.P. + 0.5 m, the normalized elevation is 0.70 in Tsuyazaki and 0.55 in Imazu. The intertidal slope is related to the sediment grain size; finer sediments accumulate in the gentle intertidal zone [47]. Thus, the communities and biomass of intertidal organisms are related to the intertidal slope [47,48], suggesting that the distribution of juvenile *T*. *tridentatus* would also be affected by the slope of the intertidal flats. Therefore, a slope raster (Fig 3) was created from the DSM using the Slope tool of Spatial Analyst in ArcGIS 10.6.

The home range of juvenile *T*. *tridentatus* has been reported to vary between 269 and 462 m^2^ [49]. Therefore, environmental conditions around the presence/absence points of the juveniles were considered to affect their distribution. The mean values of elevation, normalized elevation, and slope were calculated within 5, 10, 15, 20, 25, and 30 m around each raster cell. The six types of raster data for each variable were created using the Focal Statistics tool of Spatial Analyst in ArcGIS 10.6.

### Prior analysis

During the surveys by the first, second, and third authors at Tsuyazaki between 2018 and 2019, 50 presence points and 110 absence points of juvenile *T*. *tridentatus* were obtained. Of these absence points, three were excluded from the analysis because the ⊿Elevation exceeded 30 cm (Fig 2). Out of the 64 GCPs, 45 were set in the intertidal zones, and the juveniles were observed at one point (S2 Fig); thus, 54 GCPs were used as absence points. Accordingly, 50 presence and 161 absence points were regarded as “Training data” for HSM construction.

In the surveys by the fifth and sixth authors at Tsuyazaki in 2017, 55 presence points of juvenile *T*. *tridentatus* were obtained. Of these, 3 points were excluded because juveniles at each of these points were recaptured; thus, 52 presence points were regarded as “Test data 1.” Moreover, 60 presence points of the juveniles were obtained in the surveys conducted by the seventh author at Tsuyazaki between 2018 and 2019; thus, these 60 presence points were regarded as “Test data 2.”

During the surveys by the first and third authors at Imazu in 2019, 59 presence points of juvenile *T*. *tridentatus* were obtained. In addition, 46 of the 64 GCPs were set in intertidal zones, and the juveniles were observed at 7 points. Therefore, 59 presence and 39 absence points were regarded as “Test data 3.” Three test datasets were used for the external evaluation of HSM. The spatial distribution of the points used as Training data, Test data 1, Test data 2, and Test data 3 are shown in Fig 1 and S1 File.

**Fig 4:**
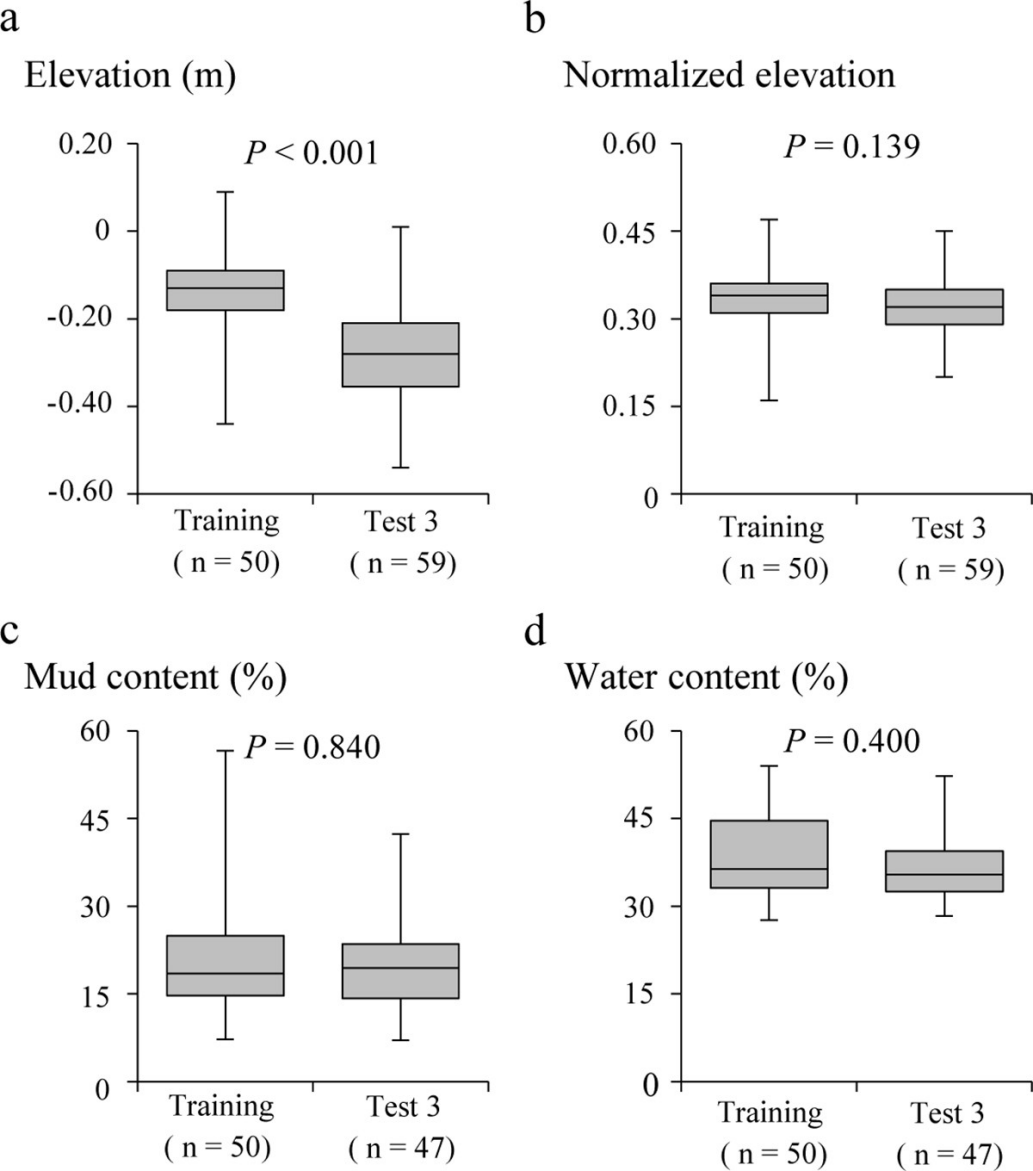
Environmental characteristics of points with detected presence of juvenile horseshoe crab in Tsuyazaki (only Training data) and Imazu (Test data 3): elevation (a), normalized elevation (b), mud content (c), and water content (d). Box plots show the 25^th^ and 75^th^ percentiles and the median. The vertical lines extend to the minimum and maximum values. P-values were based on the result of the Mann–Whitney U test.

At the presence points of Training data and Test data 3, the elevation and sediment characteristics were measured. A comparison of the four variables of elevation measured using RTK-GNSS, normalized elevation, mud content, and water content, for Tsuyazaki and Imazu, showed that only the elevation differed significantly (Fig 4). The results of the prior analysis showed that the sediment condition of the presence points was similar between the two regions. Furthermore, the results showed that the difference in the elevation between the two regions was calibrated by the tidal range. Therefore, the normalized elevation, instead of elevation, was used in the HSM construction.

### HSM creation

HSMs aimed at conservation planning should be constructed using a few variables that can be easily measured [50,51]. In the present study, the HSM of juvenile *T*. *tridentatus* was constructed using two predictor variables, normalized elevation and slope.

Generalized linear models (GLMs) were applied using the presence/absence data from the Training data as dependent variables (Binomial distribution) and the normalized elevation and slope as predictor variables. The square value of each factor was also used as a predictor variables. Null to full models were constructed according to the stepwise method, and the model with the lowest Akaike information criterion [52] was regarded as the best model. Seven types of the best model were constructed using normalized elevations, slopes, and their six mean values. Thus, the normalized elevation and slope for “GLM00,” “GLM05” (5 m mean normalized elevation and 5 m mean slope), “GLM10” (10 m mean normalized elevation and 10 m mean slope), “GLM15” (15 m mean normalized elevation and 15 m mean slope), “GLM20” (20 m mean normalized elevation and 20 m mean slope), “GLM25” (25 m mean normalized elevation and 25 m mean slope), and “GLM30” (30 m mean normalized elevation and 30 m mean slope) were used as the predictor variables. GLMs were applied using the package MuMIn [53] in R version 3.2.1 [54].

More than 70% of the absence points in the Training data were obtained in spring (May), when the juvenile activity was not high [23,43]. Therefore, it was possible that false absences had been included in the Training data. Consequently, in addition to GLMs, HSMs were constructed using Maxent, which is based on the maximum entropy method [55]. Maxent is a modeling technique that uses presence-only data, thus avoiding false absences. The habitat suitability for juvenile *T*. *tridentatus* was estimated using 50 presence points from the Training data and the raster datasets of normalized elevation and slope at Tsuyazaki (Fig 3). Seven types of Maxent models were constructed in the same manner as the GLM; normalized elevation and slope for “Maxent00,” “Maxent05” (5 m mean normalized elevation and 5 m mean slope), “Maxent10” (10 m mean normalized elevation and 10 m mean slope), “Maxent15” (15 m mean normalized elevation and 15 m mean slope), “Maxent20” (20 m mean normalized elevation and 20 m mean slope), “Maxent25” (25 m mean normalized elevation and 25 m mean slope), and “Maxent30” (30 m mean normalized elevation and 30 m mean slope) were used as the predictor variables.

The model accuracy was evaluated using the area under the receiver-operating characteristics curve (AUC). The value of AUC ranged from 0 to 1, with higher values implying higher model accuracy. As such, a model with 0.7 ≤ AUC < 0.8 was regarded to be acceptable, that with 0.8 ≤ AUC < 0.9 was regarded to be excellent, and that with AUC ≥ 0.9 was regarded to be outstanding for discrimination [56]. For all the best models, the AUC, sensitivity, specificity, and cut-off value for judging presence/absence were calculated.

To evaluate the robustness of the constructed HSMs, the models with the highest sensitivity and AUC were selected from among GLM00–GLM30 and Maxent00–Maxent30, respectively. Next, these models were evaluated using the three test datasets. Test data 1 and Test data 2 only had presence points; thus, the robustness of the HSMs was evaluated by calculating the sensitivity for each test dataset. In these two datasets, the latitude and longitude might have been less accurate than those in the Training data and Test data 3 because the measurements in Test data 1 and Test data 2 were taken using handy GPS devices. Therefore, for these datasets, when a raster cell that exceeded the cut-off value was included within the 3 m radius from a presence point, the point was regarded as a presence-predicted point. Test data 3 included both presence and absence points, and thus the sensitivity and specificity were calculated to evaluate the robustness of the HSM.

Finally, two robust HSMs selected from the models with the highest sensitivity and the highest AUC were used for habitat suitability mapping in the Tsuyazaki and Imazu intertidal flats. After mapping the suitability, the area of suitable habitats in each region was estimated.

## Results

### HSM construction

Seven types of the best GLM were constructed using the Training data for Tsuyazaki (Table 1). The sensitivity of the GLMs ranged from 0.600 to 0.860, with GLM00 exhibiting the highest sensitivity, while the AUC of the GLMs ranged from 0.671 to 0.818, with GLM05 exhibiting the highest AUC.

**Table 1:** Summary of seven generalized linear models (GLMs) with the lowest Akaike information criterion.

Model name	AUC	Cut-off value	Sensitivity	Specificity
GLM00*	0.790	0.266	0.860	0.627
GLM05**	0.818	0.327	0.800	0.727
**GLM10**	0.807	0.321	0.800	0.708
**GLM15**	0.792	0.285	0.820	0.658
**GLM20**	0.761	0.334	0.740	0.720
**GLM25**	0.694	0.310	0.600	0.665
**GLM30**	0.671	0.277	0.720	0.565

*, the highest sensitivity model

**, the highest AUC model.

The sensitivities and AUCs of the seven types of Maxent models constructed using only the presence points of the Training data were in the ranges of 0.700–0.820 and 0.780–0.804, respectively (Table 2). The model with the highest sensitivity was Maxent20, and that with the highest AUC was Maxent15.

**Table 2:** Summary of seven Maxent models.

Model name	AUC	Cut-off value	Sensitivity	Specificity
**Maxent00**	0.780	0.476	0.740	0.708
**Maxent05**	0.804	0.506	0.720	0.807
**Maxent10**	0.802	0.496	0.760	0.776
Maxent15**	0.805	0.488	0.740	0.733
Maxent20*	0.794	0.426	0.820	0.621
**Maxent25**	0.786	0.483	0.720	0.683
**Maxent30**	0.783	0.477	0.700	0.621

*, the highest sensitivity model

**, the highest AUC model.

### Selection of robust HSMs

Test data 1, Test data 2, and Test data 3 were used to evaluate the robustness of the models with the highest sensitivity (GLM00 and Maxent20) and the models with the highest AUC (GLM05 and Maxent15) (Table 3). GLM00 showed higher sensitivity than Maxent 20, with values greater than 0.7 for all three test datasets. Test data 1 and Test data 2 showed low sensitivity, between 0.517 and 0.649, for both models with the highest AUC. However, Test data 3 showed high values of both sensitivity and specificity (> 0.7) for GLM05. Therefore, GLM00 and GLM05 were considered as the robust models in the present study.

**Table 3:** Sensitivity (Sen) and specificity (Spe) of the highest sensitivity model and highest AUC model.

	Number of points		Highest sensitivity				Highest AUC			
			GLM00		Maxent20		GLM05		Maxent15	
	Pre	Abs	Sen	Spe	Sen	Spe	Sen	Spe	Sen	Spe
**Training data**	50	161	0.860	0.627	0.820	0.621	0.800	0.727	0.740	0.733
**Test data 1**	52		0.981		0.754		0.538		0.649	
**Test data 2**	60		0.733		0.683		0.517		0.550	
**Test data 3**	59	39	0.881	0.590	0.644	0.641	0.729	0.718	0.424	0.769

Pre and Abs are abbreviations for Presence and Absence, respectively.

**Fig 5:**
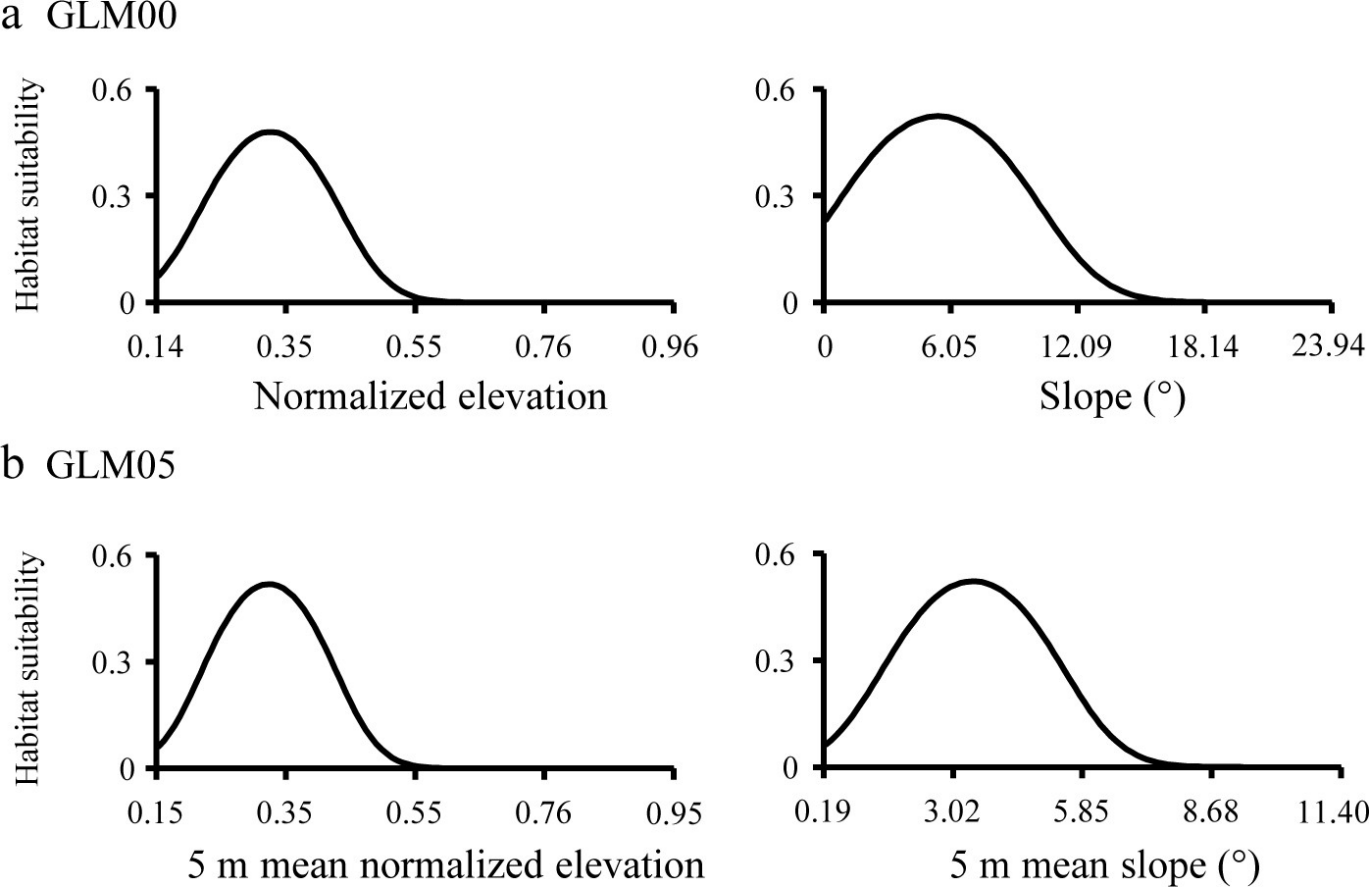
Relationships between habitat suitability of juvenile horseshoe crabs and predictor variables used for GLM00 (a) and GLM05 (b). The range of values for the predictor variables is based on the minimum to maximum values of the Training data. The other predictor variables were kept constant by assigning the median values in sites where the juveniles appeared in Tsuyazaki.

Normalized elevation, slope, and their squared variables were selected in the robust HSMs, i.e. GLM00 and GLM05 (Table 4). Each factor showed unimodal curves (Fig 5). These results indicate that low intertidal zones (normalized elevation < 0.5) with low slopes are suitable for juvenile *T*. *tridentatus*.

**Table 4:** Coefficient (Coe) and standard error (SE) of intercept and predictor variables.

Model name	Intercept		Normalized elevation		Normalized elevation^2		Slope		Slope^2	
	Coe	SE	Coe	SE	Coe	SE	Coe	SE	Coe	SE
**GLM00**	-8.68**	2.97	48.38**	17.93	-76.05**	25.77	0.49^+^	0.28	-0.05	0.03
**GLM05**	-12.60***	3.48	61.00**	19.30	-94.89***	28.37	1.83*	0.80	-0.27*	0.12

^+^, *P* < 0.1

*, *P* < 0.05

**, *P* < 0.01

***, *P* < 0.0001.

### Estimation of habitat suitability areas

**Fig 6:**
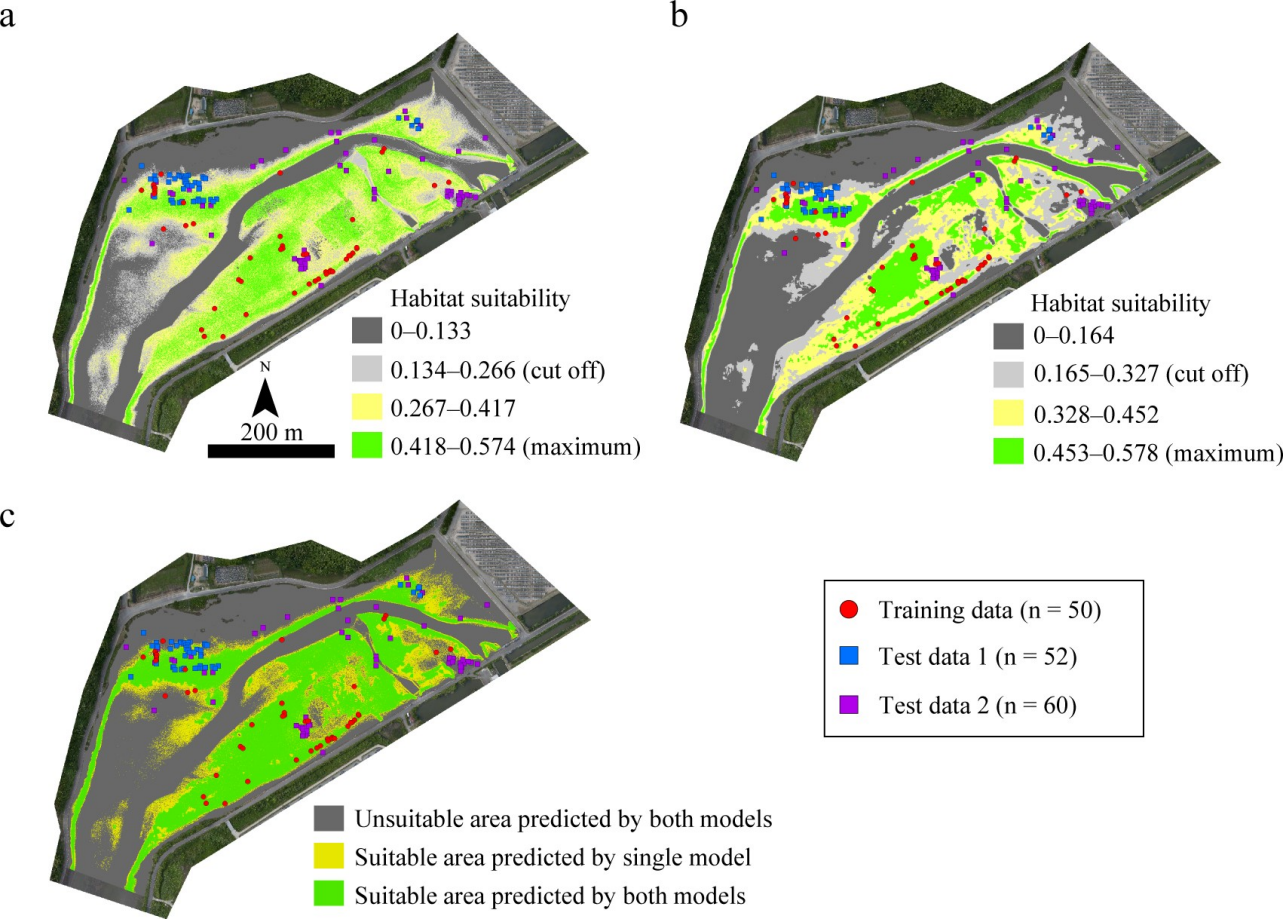
Habitat suitability maps for juvenile horseshoe crabs as predicted by GLM00 (a) and GLM05 (b) and both models (c) in Tsuyazaki intertidal flats. The orthophotos were constructed using the SfM based on aerial photographs taken by us.

The habitat suitability for juvenile *T*. *tridentatus* in approximately 19.5 ha of Tsuyazaki was estimated on the basis of each model (Fig 6). Raster cells over the cutoff value were predicted as suitable. GLM00 estimated approximately 8.8 ha (approximately 45% of the overall area) as suitable, whereas GLM05 estimated approximately 6.5 ha (approximately 34%) (Fig 6). The overlapping suitable areas predicted by both models was approximately 6.1 ha (Table 5 and Fig 6).

**Table 5:** Correspondence of suitable and unsuitable areas (ha) between GLM00 and GLM 05 in Tsuyazaki.

		GLM05		Total
		Suitable area	Unsuitable area	
**GLM00**	**Suitable area**	6.1	2.7	8.8
	**Unsuitable area**	0.4	10.3	10.7
	**Total**	6.5	13.0	

**Fig 7:**
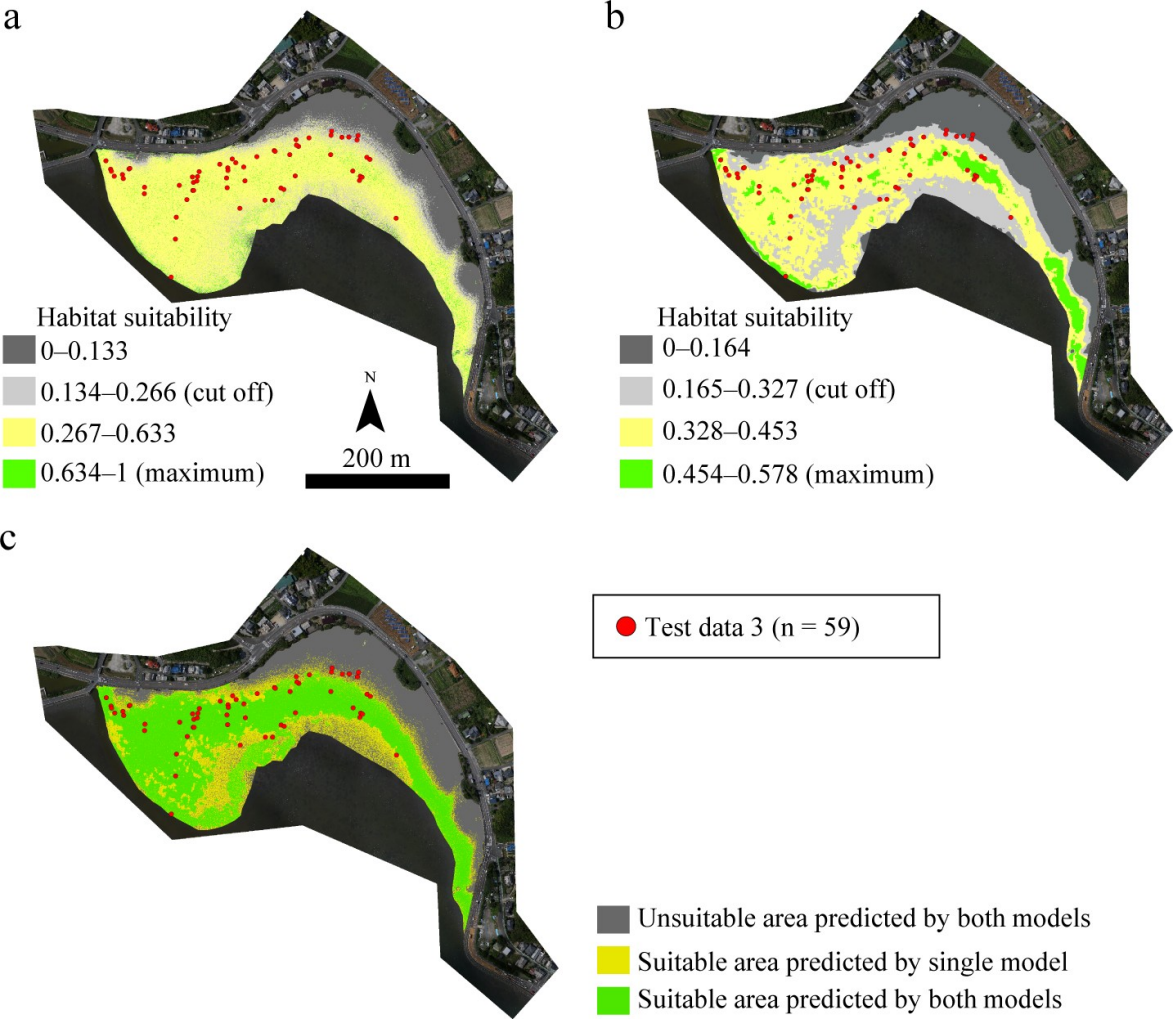
Habitat suitability maps for the juvenile horseshoe crabs as predicted by GLM00 (a) and GLM05 (b) and both models (c) in Imazu intertidal flats. The orthophotos were constructed using the SfM based on aerial photographs taken by us.

The HSMs (GLM00 and GLM05) extrapolated the habitat suitability area in approximately 7.9 ha of Imazu, showing suitable areas of approximately 5.2 ha (approximately 66% of the overall areas) and approximately 4.0 ha (approximately 51%), as shown in Fig 7. The overlapping suitable area extrapolated by both models was approximately 3.7 ha (Table 6 and Fig 7).

**Table 6:** Correspondence of suitable and unsuitable areas (ha) between GLM00 and GLM 05 in Imazu tidal flat.

		GLM05		Total
		Suitable area	Unsuitable area	
**GLM00**	**Suitable area**	3.7	1.5	5.2
	**Unsuitable area**	0.3	2.4	2.7
	**Total**	4.0	3.9	

**Fig 8:**
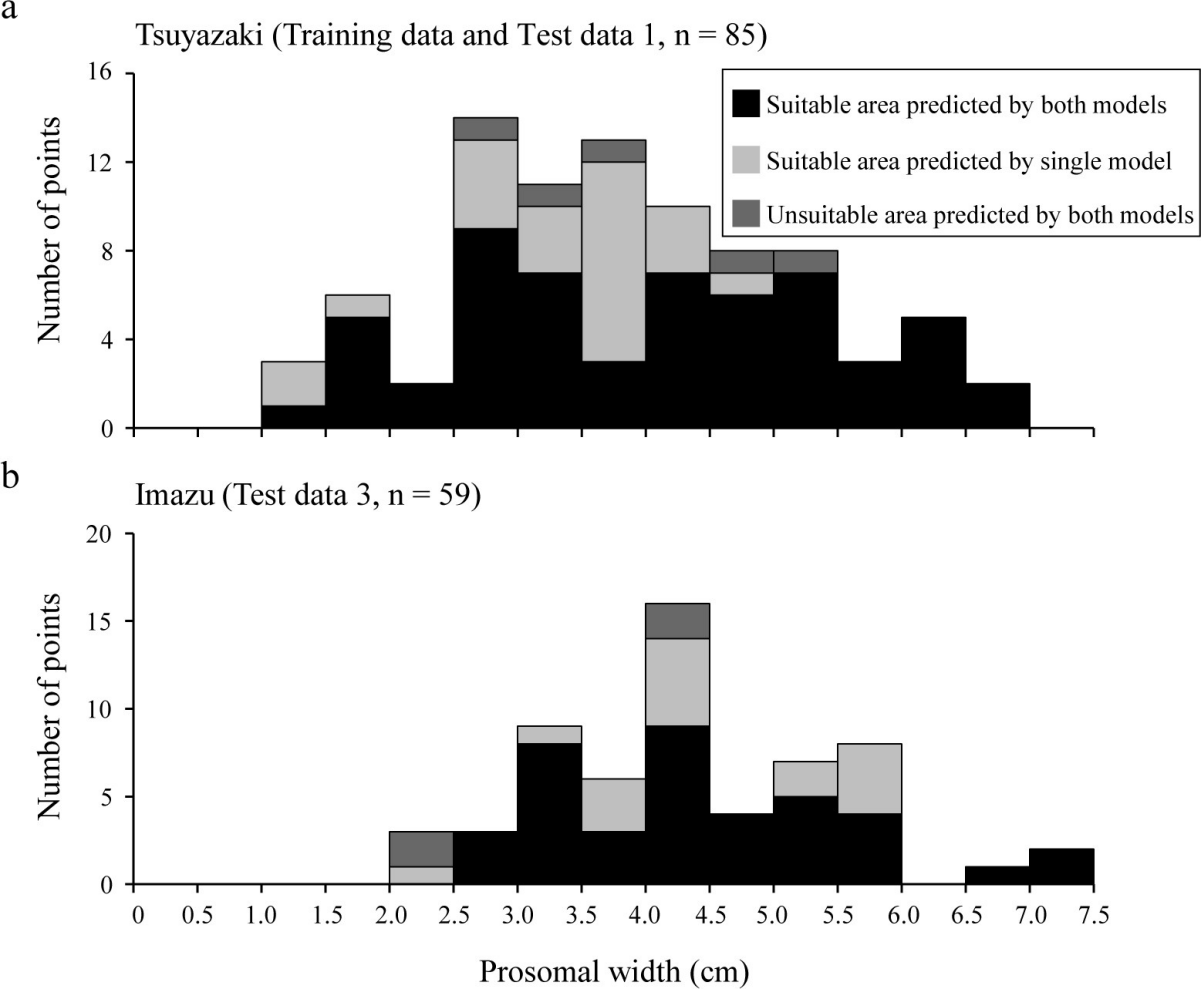
Frequency of prosomal width among suitable and unsuitable areas as predicted by CLM00 and GLM05 in Tsuyazaki (a) and Imazu (b).

Finally, to confirm whether the habitat suitability area covered all sizes of juvenile *T*. *tridentatus*, relationships between habitat suitability and prosomal width were determined for each region (Fig 8). Test data 2 was excluded because the prosomal width was not measured for this dataset. At Tsuyazaki, 57 of the 85 presence points (approximately 67%) were plotted within overlapping suitable areas, and this area covered all size classes. In Imazu, 39 of 59 presence points (approximately 66%) were plotted in overlapping suitable areas, and this area covered all size classes except those less than 2.5 cm.

## Discussion

### Evaluation of HSM

As mentioned earlier, an HSM for juvenile *T*. *tridentatus* at Tsuyazaki using UAV-SfM has also been constructed by Iyooka [34]; however, neither an internal nor external evaluation of the HSM was performed, and thus the robustness of the model was not verified. In the present study, three test datasets were used as external data to evaluate the HSMs with the highest sensitivity and AUC. The GLMs showed relatively lower specificity than sensitivity (Table 1). This may be due to the presence of certain false absence points included in the Training data. However, the AUCs of these models, except of GLM25 and GLM30, were more than 0.7; thus, the negative influence of false absences was small in GLM construction. The sensitivity of Maxent20 and Maxent15 to Test data 3 was low (Table 3), suggesting that these models, constructed using only the presence data, were overfitting to the training dataset [57]. In contrast, GLM00 showed a high sensitivity to all three test datasets. GLM05 showed low sensitivities to Test data 1 and Test data 2, but high sensitivity and specificity to Test data 3 (Imazu). Furthermore, although no juveniles were recorded in survey area T2 at Tsuyazaki in the Training data surveys, both GLM00 and GLM05 predicted suitable areas in T2, and several juveniles were found there in the surveys used for Test data 1 and Test data 2 (Fig 6). Therefore, to the best of our knowledge, this study is the first to suggest that a robust HSM can be constructed using DSMs created with the UAV-SfM technique and slopes calculated based on the DSM.

The effective variables of HSM can explain the habitat characteristics of the target species. Iyooka [34] used elevation and intertidal slope to construct the HSM but did not explain the importance of these factors for juvenile *T*. *tridentatus*. The juveniles feed in intertidal areas with 1–10 cm of surface water at low tides [58]. In the present study, most juveniles appeared in the lower intertidal zones (0 < normalized elevation < 0.5), and the response curves of the normalized elevation peaked at approximately 0.3 (Figs 4 and 5). Thus, the response curves of normalized elevation in our GLMs correspond to the results of previous studies. Intertidal zones with a surface slope less than 1/100 (i.e., < 0.3°) are considered as tidal flats [47]. However, the habitat suitability peaked at a slope of approximately 5.3° in GLM00 and at a mean slope of approximately 3.4° in GLM05 (Fig 5); thus, these values are higher than those previously reported. In the present study, the raster cell size was set as 0.2 m. If the difference in elevation among eight adjacent raster cells was 0.04 m, the slope was calculated between 2.02° to 6.38° according to the Slope tool in ArcGIS. We consider that such small asperities on the surface indicate the existence of several tidal pools used by the juveniles during low tide. The intertidal flat clearly has a very low slope, but our GLMs suggest that the surface slope may affect the distribution of estuarine organisms on a small spatial scale.

For conservation planning, the ideal HSM should have a high predictive performance [50,51]. The accuracy of our HSMs was not outstanding, but it was acceptable or excellent in discrimination (Table 1). This may be attributed to the lack of important factors explaining the habitat characteristics of the target species [59]. For example, biotic and abiotic factors, such as sediment condition, density of prey, and coverage of seagrass patches, affect the distribution of the juveniles [20,21,60]. The sediment condition, which is one of the factors affecting juvenile distribution, can be estimated with further development of the UAV-SfM technique [31]. Therefore, we consider that our models can predict the habitat suitability of the juveniles, but there is room for improvement.

### Habitat suitability areas

Drafting conservation management plans for *T*. *tridentatus* should consider genetic subdivisions [12]. The species inhabiting Tsuyazaki and Imazu have similar genetic structures, and these two regions are under one local management unit in Japan [61]. Thus, the current habitat suitability maps of both these regions can provide basic information for drafting conservation plans under this local unit. The intertidal habitats in Japan are less developed than on the continent because the Japanese islands are small and mountainous. Therefore, long term monitoring of the increase/decrease in the small intertidal habitats by mapping of the spatial distribution can be expected to contribute to adaptive management for juvenile conservation.

The total habitat suitability area within each region, as obtained by GLM00 and GLM05, differed by more than 10 percentage points (Tables 5 and 6). GLM00 showed a higher sensitivity to the three test datasets than GLM05 (Table 3). This suggests that GLM00 is the best model for estimating the habitat suitability area for the juveniles. However, the area estimated as suitable for habitat by GLM00 was more than 1.3 times the area deemed suitable by GLM05. Furthermore, the specificities of Training data and Test data 3 were lower for GLM00 than for GLM05 (Table 3). These results indicate the possibility that GLM00 might be overestimating the suitable areas. Therefore, in the present study, it is reasonable to consider the overlapping areas as suitable habitats, as approximately 70% juveniles appeared in those areas. Accordingly, the current habitat suitability areas are at least 6.1 ha in Tsuyazaki and at least 3.7 ha in Imazu (Tables 5 and 6).

Although the size of juvenile *T*. *tridentatus* observed in intertidal zones varies among regions [22,23,43,62], previous studies reported that the juveniles of 1.4 to 7.0 cm appeared in intertidal zones of Kitsuki City [13], and those of 8.05 to 76.00 mm appeared in the Tsuyazaki intertidal flats [39]. The size ranges of the juveniles recorded in our surveys generally agree with previous Japanese reports, and they were estimated to be in the third to eleventh instar stages [14,22]. In addition, all classes of prosomal width were covered in the overlapping suitability areas in Tsuyazaki (Fig 8). These results suggest that our HSMs can represent suitable habitats for juveniles in the third to eleventh instar stages that inhabit intertidal flats in Japan. However, juveniles with less than 2.5 cm prosomal width were not recorded in the overlapping suitability areas in Imazu (Fig 8). Chiu and Morton [43] have reported that larger juveniles tend to appear offshore, i.e., at lower elevations in the intertidal zone. Thus, it should be noted that habitat suitability areas for the juveniles in each instar stage may be more localized than those shown in our maps.

In both regions, the upper intertidal zones adjacent to the road and the subtidal zones are unsuitable habitats for juvenile *T*. *tridentatus* (Figs 6 and 7); however, our results do not imply that these zones are unnecessary environments for horseshoe crabs. In Tsuyazaki, the adults inhabit seagrass beds approximately 2 km offshore from our survey areas [38]. The juveniles are seldom active during high tide [13], and thus, the rill in the central part of the Tsuyazaki intertidal flats, i.e., the subtidal zones, may serve as a pathway for larger juveniles to migrate offshore during low tide. Therefore, for the conservation of the local population, it is essential to maintain sufficient water depth in the rill so the juveniles can migrate at low tide. In the survey area in Imazu, the sandy beaches of the upper intertidal zones adjacent to a road are the most important spawning sites of this species in the Hakata Bay [14,42]. Because this species inhabits various estuarine habitats throughout its life cycle, maintaining connectivity between these habitats is essential for conserving the local populations [12]. In the future, studies should be conducted to estimate suitable areas where adults can live and spawn.

Moreover, while our study covers most intertidal areas in Tsuyazaki inhabited by juveniles, it did not cover several intertidal flats in Imazu, which may be inhabitable for *T*. *tridentatus*. We consider that habitat suitability maps of these areas may be created by the UAV-SfM technique, but further investigation is essential for their verification.

## Conclusion

Although several issues remain regarding the construction of the HSMs, our findings show the effectiveness of the UAV-SfM technique in estimating habitat suitability areas for intertidal species such as *T*. *tridentatus*. In particular, the normalized elevation and slope, which are used as predictor variables, can be assessed over a wide area using the UAV-SfM technique and GIS tools. In this manner, these variables can be collected more easily and at lower costs than multiple surveys that include the measurements of environmental variables, such as salinity, dissolved oxygen, sediment condition, and biotic components. Changes in habitat suitability areas due to anthropogenic activities or natural disturbances, such as typhoons, can be easily estimated by recreating the topographical models using the UAV-SfM technique. For the conventional monitoring of juveniles, many investigators are required, thus posing the risk of squashing the juveniles and destroying their habitats [39]. Using the UAV-SfM technique, approximately 20 ha of the Tsuyazaki intertidal flats could be surveyed by just two or three investigators. Therefore, the UAV-SfM technique is expected to reduce the number of investigators and also the associated risk.

The population size could not be estimated using our HSMs because they were constructed on the basis of presence/absence data. For local conservation management, Wang et al. [63] proposed a framework and minimum standards to evaluate baseline information on juvenile *T*. *tridentatus* populations. Consequently, they recommended quadrat methods along with transect sampling to obtain abundance and density data of the juveniles. They also emphasized the mapping of environmental characteristics and the juvenile population in the entire survey area. We expect that the UAV-SfM technique and HSMs based on the population or density data will strengthen their framework [63]. Therefore, further studies focused on the UAV-SfM technique will be required to consolidate long-term monitoring methods for local populations of the juvenile *T*. *tridentatus*.

## Supporting information

S1 Fig Spatial distribution of juvenile horseshoe crabs collected by the first, second, and third authors. Each plot shows the survey date and time at each area. The orthophotos were constructed using the SfM based on aerial photographs taken by us.
(TIF)

S2 Fig Spatial distribution of ground control points (GCPs) and ground references (GRs) in the Tuyazaki and the Imazu intertidal flats. The orthophotos were constructed using the SfM based on aerial photographs taken by us.
(TIF)

S3 Fig Measurement points of the rill (subtidal zone) in Tsuyazaki (a), elevation estimated by the digital surface model (DSM) (b), and corrected DSM based on measurement points (c). The points were measured by the Real time kinematic-global navigation satellite system (RTK-GNSS), and DSM was created by the UAV-SfM. Corrected DSM were created using the Spatial Analysist tool of the ArcGIS software. The orthophotos were constructed using the SfM based on aerial photographs taken by us.
(TIF)

S1 File Raw dataset used for HSMs constructing and external validation. This file contains Training data, Test data 1, Test data 2, and test data 3.
(XLSX)

S2 File Digital surface models of Tsuyazaki and Imazu. This raster data are used for HSM construction.
(ZIP)

S3 File (PDF)
