# Supplementary figures and images for: Habitat suitability maps for juvenile tri-spine horseshoe crabs in Japanese intertidal zones: A model approach using unmanned aerial vehicles and the Structure from Motion technique
Source: PLoS One. 2020 Dec 23;15(12):e0244494. doi: 10.1371/journal.pone.0244494 (PMC7757885; doi:10.1371/journal.pone.0244494)

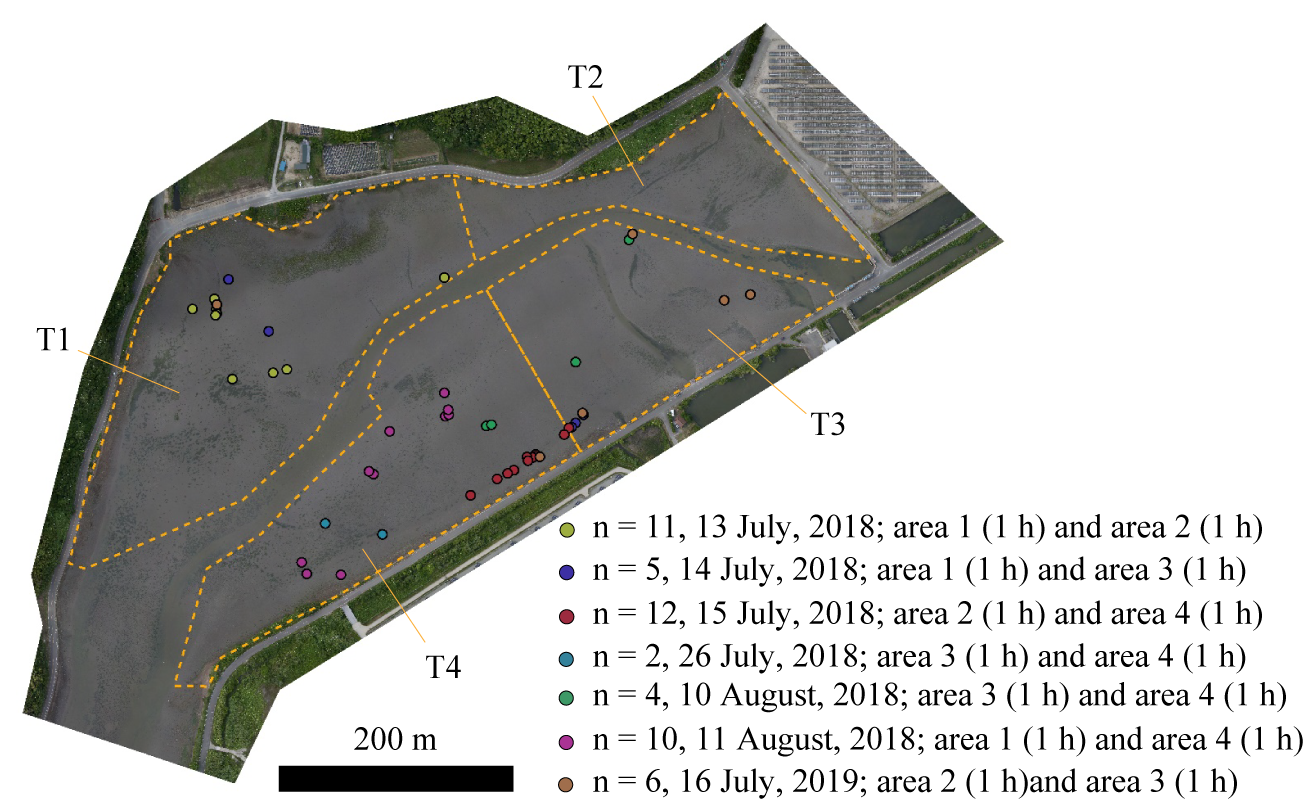

Supplement: S1 Fig — Each plot shows the survey date and time at each area. The orthophotos were constructed using the SfM based on aerial photographs taken by us. (TIF) [file pone.0244494.s001.tif]

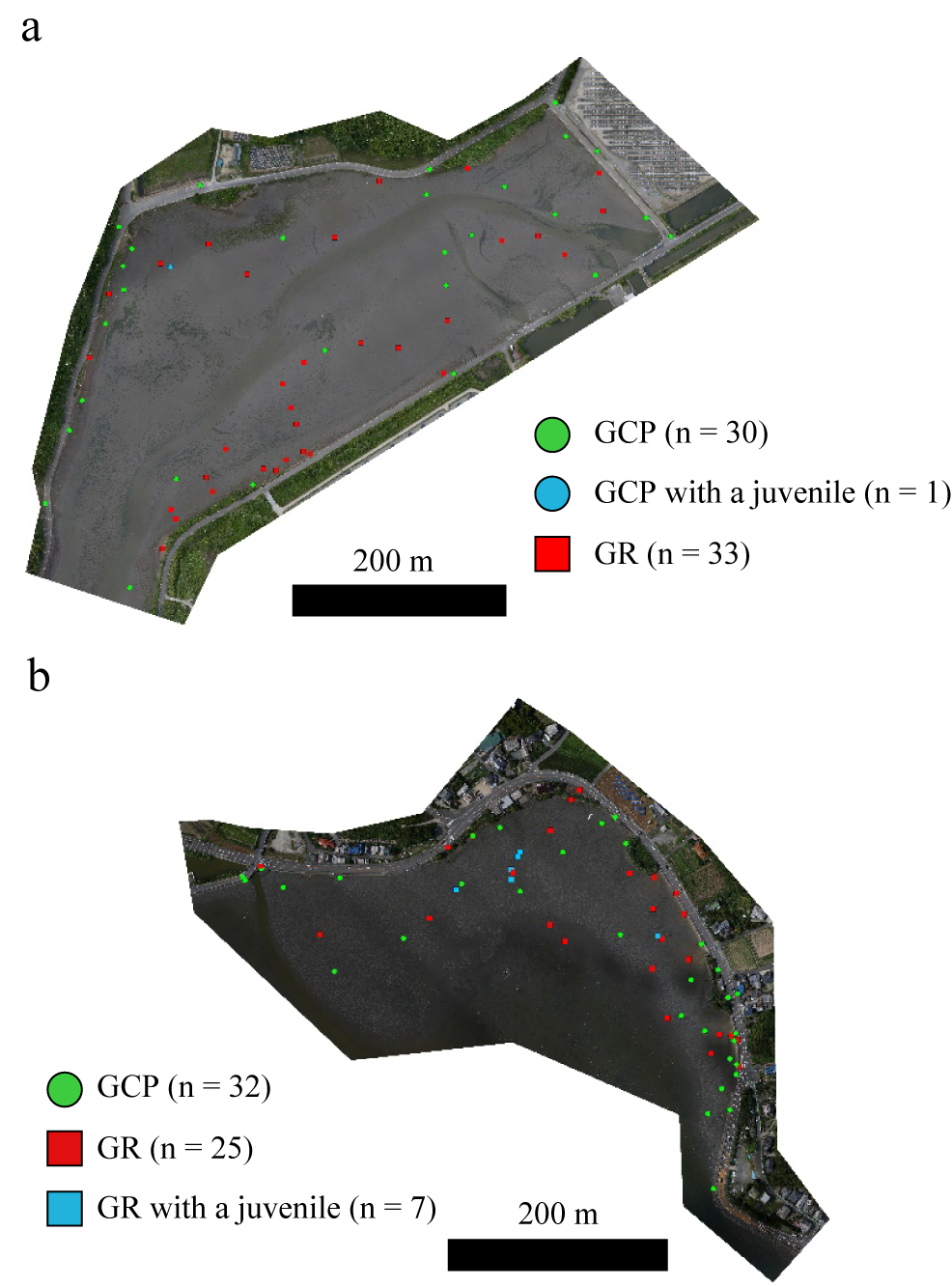

Supplement: S2 Fig — The orthophotos were constructed using the SfM based on aerial photographs taken by us. (TIF) [file pone.0244494.s002.tif]

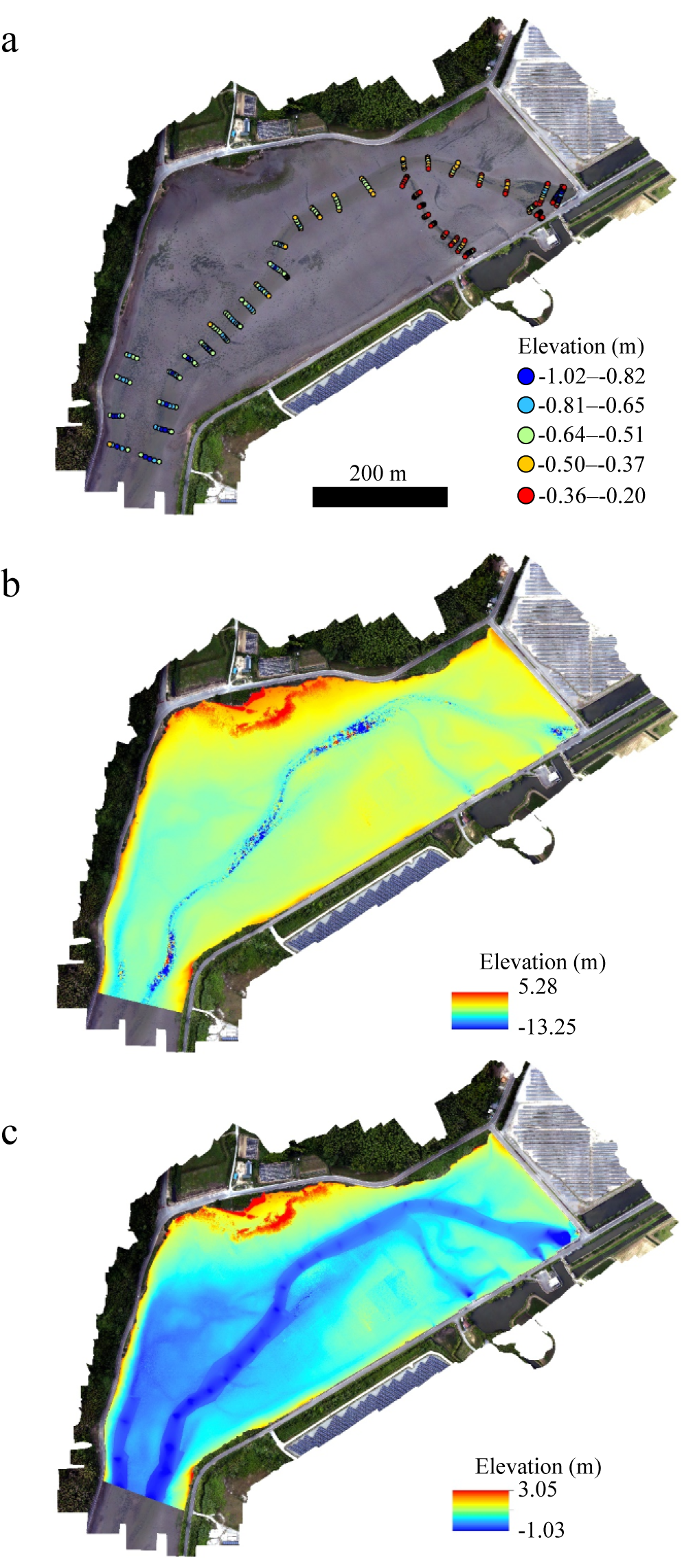

Supplement: S3 Fig — Measurement points of the rill (subtidal zone) in Tsuyazaki (a), elevation estimated by the digital surface model (DSM) (b), and corrected DSM based on measurement points (c). The points were measured by the Real time kinematic-global navigation satellite system (RTK-GNSS), and DSM was created by the UAV-SfM. Corrected DSM were created using the Spatial Analysist tool of the ArcGIS software. The orthophotos were constructed using the SfM based on aerial photographs taken by us. (TIF) [file pone.0244494.s003.tif]
